# Role of Using Nonsteroidal Anti-Inflammatory Drugs in Chemoprevention of Colon Cancer in Patients With Inflammatory Bowel Disease

**DOI:** 10.7759/cureus.8240

**Published:** 2020-05-22

**Authors:** Lamis F Abdalla, Reem Chaudhry Ehsanullah, Fazida Karim, Azeezat A Oyewande, Safeera Khan

**Affiliations:** 1 Internal Medicine, California Institute of Behavioral Neurosciences and Psychology, Fairfield, USA; 2 Psychology, California Institute of Behavioral Neurosciences and Psychology, Fairfield, USA; 3 Business & Management, University Sultan Zainal Abidin, Terengganu, MYS; 4 Family Medicine, California Institute of Behavioral Neurosciences and Psychology, Fairfield, USA; 5 Family Medicine, Lagos State Health Service Commission/Alimosho General Hospital, Lagos, NGA

**Keywords:** ibd, colitis associated cancer, colorectal cancer, nsaids, mesalazine, chemoprevention

## Abstract

The process of inflammation occurs due to inflammatory mediators, including prostaglandins, cytokines, and tumor necrosis factor (TNF). All these mediators activate the process of tumorigenesis and dysplasia, leading to colitis-associated cancer. Several drugs used to decrease these mediators will help in the treatment of acute attacks and also help in prolonged remissions of the disease by using nonsteroidal anti-inflammatory drugs (NSAIDs), steroids, and biological factors. Reducing these inflammatory mediators also have a role in chemoprevention and prevent progression to colorectal carcinoma. The most researched drugs in this process of chemoprevention are NSAIDs as it has both cyclooxygenase-2 (COX-2) inhibitory and non-inhibitory effects. These drugs should be taken for a long time and in large doses to reach this effect, which puts the patient at risk for various side effects. Researchers will need to do more research in the future to find the lowest effective dose that can reach the chemopreventive effect. We used database Pubmed as the main source for data search and extracted articles exploring the relationship between NSAIDs and their role in chemoprevention of colorectal carcinoma in inflammatory bowel disease (IBD) patients. We chose 23 studies which included seven review articles. We found that inflammatory mediators have a key role in colitis-associated cancer.

## Introduction and background

Inflammatory bowel disease (IBD) is a broad term that describes a group of conditions that affect the gastrointestinal system causing inflammation and several other changes to the mucosa in ulcerative colitis (UC) or all layers of intestines in Crohn's disease. All these changes can occur because of interactions between environmental and genetic factors and microbial, leading to immunological response and inflammation of the intestine [1].

In 2015, an estimated 1.3% of adults (3 million) reported being diagnosed with IBD either UC or Crohn's. The estimate does not include children under 18 years, who may also have IBD, as most people with IBD usually diagnosed in their 20-30 according to Center of Disease Control and Prevention (CDCP) data and statistics [2].

IBD is a fundamental cause of colon cancer as the relation between inflammation and dysplasia is well established, mainly when diagnosed at an early age, for longer duration and extensive involvement of the colon. Some of the fundamental stages in cancer development is the formation of aberrant crypt foci, polyps, adenoma, and carcinoma [3]. That's why screening with colonoscopy should be started regularly for all patients with IBD 8-10 years after diagnosis, to include random four quadrants biopsies every 10 cm [4,5].

The risk of colon cancer is increased by at least two folds in UC compared to healthy populations. Colon cancer is observed in 5.5% to 13.5 % of all patients with UC, and 0.4% to 0.8% inpatient with Crohn's disease [6].

**Figure 1:**
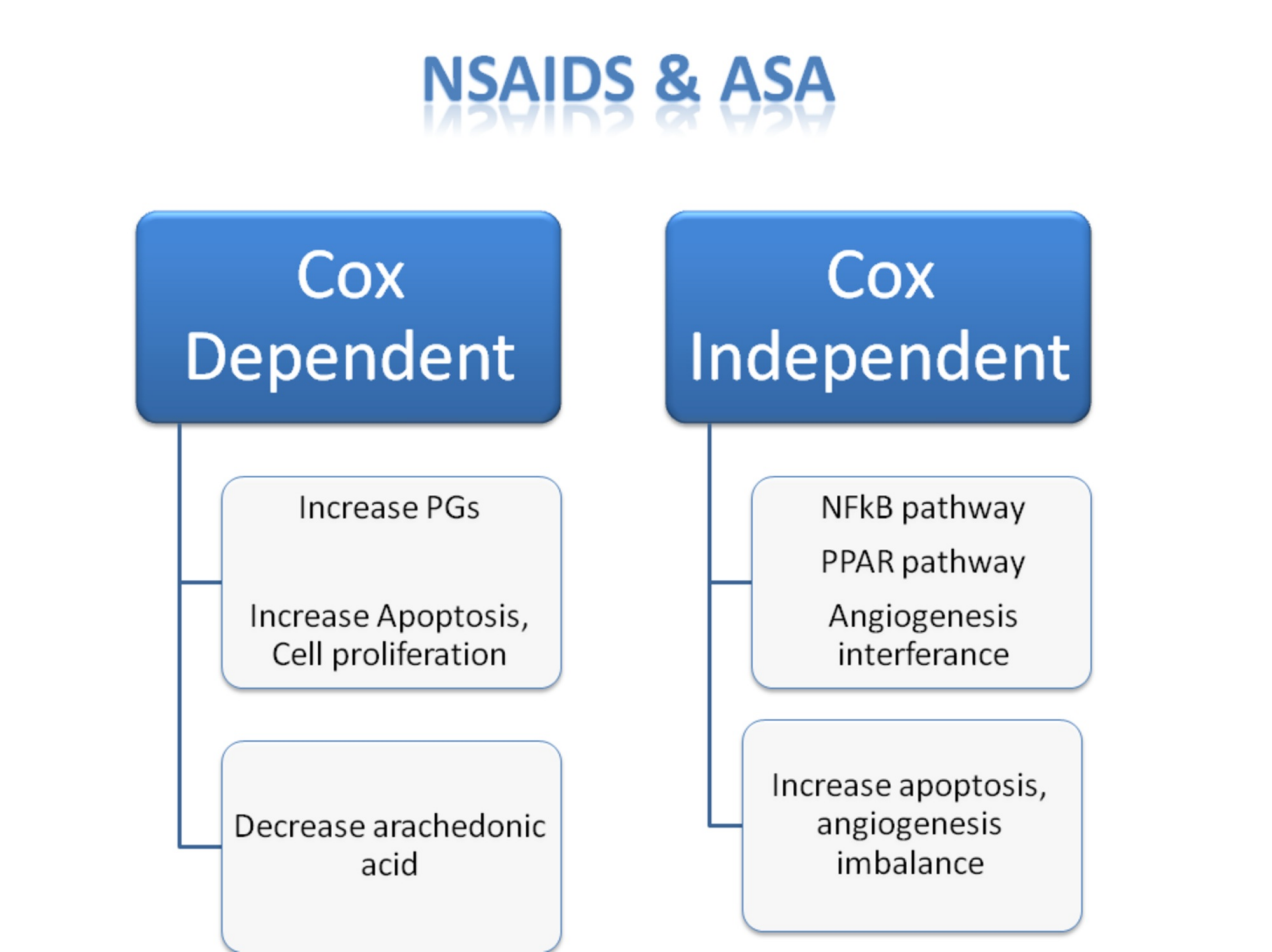
Comparison between COX-2 dependent and COX-2 independent effects of NSAIDs COX-2, cyclooxygenase-2; NSAIDs, nonsteroidal anti-inflammatory drugs; PGs, prostaglandins, NF-kB, nuclear factor Kappa light chain enhancer of activated B cells; PPAR, peroxisome proliferator-activated receptor

Recently a relation is found between using aspirin and decreasing incidence of colorectal carcinoma. Using aspirin as a chemopreventive method is due to its inhibitory and non-inhibitory effect on cyclooxygenase-2 (COX-2) and its antiplatelet effect, which all help in decreasing cell proliferation, and the established dysplasia in the cell, leading to lower risk of colorectal carcinoma (CRC) by 24% and reduce cancer-associated mortality by 35% after a delay of 8-10 years as shown in Figure 1 [7,8].

In this study, we aim to review the relationship between using NSAIDs, aspirin, 5-aminosalicylic acids (5-ASA), mesalazine, and sulindac in IBD and its role in decreasing colorectal carcinoma incidence in IBD patients and also the mechanism of action to do that, and we also aim to clarify which one of these drugs with fewer side effects as they can lead to many complications with long-term usage. We also need to know the minimum effective dosing for chemoprevention function and the duration of using for this. We already know the importance of using these drugs to decrease inflammation in IBD especially in UC (not so effective in Crohn's disease as steroids show more effectiveness especially with adding biological factors like rituximab). Establishing this relationship will help to reduce the cancer burden for the patient, hospital, and government, aiming for the patient to lead a better life dealing and tolerating his or her chronic illness instead of having severe complications like colorectal carcinoma, which will worsen his or her life physically psychologically and economically.

## Review

Methods

The research was conducted to identify studies analyzing the effect of NSAIDs and mesalazine in decreasing colorectal cancer in patients with IBD patients. We systematically searched PubMed as the primary database to search for the relevant articles. A few other articles from within the references that explored the related concepts were also added to provide a broad understanding of the topic. Keywords used for the search were inflammatory bowel disease and complications, NSAIDS, mesalazine, colorectal carcinoma cancer-associated colitis, as shown in Table 1. We selected 23 articles; seven out of them were review articles.

**Table 1:** Methods IBD, inflammatory bowel disease; NSAIDs, nonsteroidal anti-inflammatory drugs

Keywords/combinations	Database	No. of results
IBD	Pubmed	90879
Colorectal carcinoma	Pubmed	109317
NSAIDs	Pubmed	68995
Mesalazine	Pubmed	1909
Mesalazine/IBD	Pubmed	400
IBD/colorectal cancer	Pubmed	1174
Mesalazine/IBD/colon cancer	Pubmed	36

Results

Twenty-three studies were finalized after thorough data search, seven out of those selected studies were clinical reviews (shown in Table 2), three of them evaluated the use of anti-inflammatory drugs in colon cancer chemoprevention, especially in IBD patients [9-11]. One of them discussed the role of chronic inflammation and its effect on carcinogenesis [12]. One of the studies clarified the mechanism of aspirin as a chemopreventive agent [13]. One study discussed the side effect of long-term aspirin use and how to overcome them [14]. The last one explored a new medication that helps to overcome the side effects of long-term use of NSAIDs [15].

**Table 2:** Summary of some of the included studies COX-2, cyclooxygenase-2; NSAIDs, nonsteroidal anti-inflammatory drugs; UC, ulcerative colitis; NOSH-aspirin, nitric oxide and H2S releasing aspirin; NF-kB, nuclear factor of Kappa light chain enhancer of B cells

Author/year	Type of study	Purpose of the study	Results/conclusion
Ullman and Itzkowitz, 2011	Clinical review [12]	Intestinal inflammation and cancer	Patients with UC and Crohn's disease believed to have increased risk of colon cancer as chronic inflammation promotes carcinogenesis
Tsioulias et al., 2015	RCT [11]	NSAIDs and colorectal cancer control: promise and challenges	Some people are eligible to be a candidate for chemoprevention with NSAIDs
Mohammed et al., 2018	Clinical review [9]	Clinically relevant anti-inflammatory agents for chemoprevention of colorectal cancer: new perspectives	Number of drugs have to be studied to use for chemoprevention as NSAIDs but still need more studies to reduce the side effect of the use
Chen and Stark, 2017	Clinical review [10]	Aspirin prevention of colorectal cancer: focus on NF-κB signaling and the nucleolus	Aspirin and related NSAIDs have anti-tumor activity and the potential to prevent cancer, particularly colorectal cancer
Dovizio et al., 2012	Clinical review [13]	Mechanistic and pharmacological issues of aspirin as an anticancer agent	Chemopreventive mechanism of aspirin thought to act through COX-2 inhibitory and COX-2 non-inhibitory and antiplatelet effects
Rodríguez et al., 2016	A systematic review [14]	Bleeding risk with long-term low-dose aspirin: a systematic review of observational studies	The risks of significant bleeding with low-dose aspirin in real-world settings are of a similar magnitude to those reported in randomized trials
Kodela et al., 2015	Clinical review [15]	NOSH-aspirin (NBS-1120), a novel nitric oxide- and hydrogen sulfide-releasing hybrid has enhanced chemopreventive properties compared to aspirin, is gastrointestinal safe with all the classic therapeutic indications	NOSH-aspirin (NBS-1120), a novel hybrid that releases nitric oxide and hydrogen sulfide, was designed to be a safer alternative for aspirin In chemoprevention

Discussion

IBD is a complex disease that results from many factors like environment, bacterial, and genetic [16]. All these factors will lead to loss of integrity of the gastrointestinal epithelium with the dysfunction of innate immunity and Toll-like receptors. This will cause abnormal signaling in immune responses to molecules that broadly shared by many pathogens leading to the acute and chronic inflammatory process of IBD and cancer-associated colitis [17].

There are two primary diseases of IBD, which are Crohn's disease and UC. The prevalence rate of both of them is 11,2 million worldwide, with a death rate of 47,400 per year (2015). About 1.3% of adults in the United States have IBD [2]. These two primary conditions have some differences in pathophysiology, symptoms, and complication (shown in Table 3).

**Table 3:** Comparison between Crohn's disease and ulcerative colitis

	Crohn's disease	Ulcerative colitis
Age of onset	Bimodal 15-30 years, 60-70	Bimodal, 15-30, above 60
Common sites	Terminal ileum (could be from mouth to anus)	Colon and rectum
Gross features	Focal aphthous ulcer with intervening normal mucosa, linear fissures, cobblestone appearance	Crypt abscesses
Microscopic	Non-caseating granuloma	Limited to mucosa and submucosa
Colorectal cancer risk	1-3%	5-25%
Symptoms	Crampy abdominal pain, diarrhea	Bloody diarrhea
Complications	Fistula, abscesses stricture, obstruction	Toxic megacolon
Surgery role	Treatment of complications	Curative

Being affected with IBD can limit the quality of life due to symptoms like abdominal pain, diarrhea, anemia, and other displeasing symptoms and complications. Also, life expectancy decreased for people affected with IBD due to an increased risk of cardiovascular diseases, infections, and cancers [18]. 

IBD and Colorectal Cancer

Colorectal carcinoma is the third commonest cancer worldwide in men and second-most fatal cancer in the United States, it might include sporadic cases or colitis-associated carcinoma [19].

Risk factors for sporadic cases include male sex, age more than 50, first-degree relative diagnosed with cancer colon, obesity, sedentary life consumption of a large amount of red meat, alcohol, and smoking [16]. People with UC are more vulnerable to CRC rather than those with Crohn's disease, especially if diagnosed with primary sclerosing cholangitis (PSC) (which is mostly associated with UC), that's why we should do a colonoscopy at the time of diagnosis of PSC with follow-up of one to two years intervals [16,20]. Risk factors for IBD patients to develop CRC depends on the extent, severity, and duration of colonic involvement also cases complicated with strictures, pseudo polyps, anatomical abnormalities, shorted colon, and personal history of flat dysplasia [19]. All these risk factors put the IBD patient to be 1.5 to two times more vulnerable than the healthy population [19].

Teenagers with IBD have the risk of CRC about 15% so for satisfying consequences can reach with better control of inflammation with chemopreventive drugs (like 5-ASA, mesalazine, sulindac, etc.), screening with regular colonoscopy should start after diagnosis by 8-10 years and done regularly at one to two years intervals. Colectomy can be curative for the patient with colonic involvement [21]. 

Colitis-Associated Cancer

The pathophysiology of colitis-associated cancer: Chronic inflammation incriminated in starting the process of dysplasia through increasing oxidative stress, and persistent production of growth factors as well as reactive oxygen and nitrogen species, which interact in DNA of epithelium resulting in activation of procarcinogens genes and/or inhibition of tumor suppressor genes [22,23].

In addition to the carcinogenic effect of inflammation, it also plays a vital role in dysplasia and metastatic spread due to several inflammatory mediators, which can then lead to tumor progression beyond the inner layers (like cytokines, prostaglandins, growth factors, tumor necrosis factors, and chemokines). Some of these factors act directly on the tumor, cell-stimulating their proliferation and inhibition of apoptosis. Epidemiologic studies show that about 20% of chronically inflamed cells transfer to cancerous cells as a result of chronic inflammation. Understanding this pathophysiology prompts researchers to study chemopreventive drugs further to clarify their role in decreasing the CRC in overall healthy people and particularly in IBD patients. Many studies approached preventive measures as adjuvant strategies for screening, treatment, and surgery. These studies work on adding a low dose of aspirin, 5-ASA, mesalazine, statins, folic acids, vitamin D, Ca, and antioxidants with a lack of evidence to support their immediate effect.

Role of NSAIDs as Chemopreventive Drugs

Among these methods, we found that adding a low dose of aspirin or NSAIDs act as a chemopreventive method. The mechanism of action of these medications that conceptualized in chemoprevention is COX dependant and COX independent (Table 4) [9].

**Table 4:** Comparison between COX-2-dependant and COX-2-independent effect of NSAIDs COX-2, cyclooxygenase-2; NSAIDs, nonsteroidal anti-inflammatory drugs; PGE, prostaglandins E; Wnt, signaling pathway; NF-kB, nuclear factor Kappa light chain enhancer of activated B cells; P53, cellular tumor antigen; NOS-2, nitric oxide synthase 2; P21, potent cyclin dependant kinase inhibitor; STAT 3, signaling transducer and activator of transcription; TNF, tumor necrosis factor; IL, interleukin, AMPK, adenosine monophosphate protein kinase; PCNA, proliferating cell nuclear antigen; p65, transcription factor of p 65

COX-dependent effect	COX-independent effect
Catalyze the synthesis of prostaglandins and PGE2, which stimulate proliferation of cancer cells, inhibit apoptosis, act as pro-inflammatory and immunosuppressive and stimulate tumor angiogenesis with dose 81-325 mg per day	-wnt, β-catenin, NF-κB, p53, toll-like receptor 4, NOS-2, and caspases for (aspirin). -p21, p53, Wnt, NF-κB, STAT3, TNF-α, IL-1β, IL-4, AMPK, PCNA, cyclin D1, β-catenin, inducible NOS, COX-2, and p65 (for sulindac with dose 300 mg per day)

The anticancer effect of NSAIDs was initially considered only to be due to COX inhibitory effect but later found to be due to both COX inhibitory and COX non-inhibitory effects. COX inhibitory pathway involves Prostaglandins. When the enzyme catalyzes the synthesis of PGE2 (prostaglandins), it stimulates the proliferation of the cancer cells. Hence, the inhibition of COX-2 leads to the inhibition of growth and induction of apoptosis in tumor cells. However, extra work is done with COX-independent effects, which was found to help significantly in the chemopreventive role.

**Figure 2:**
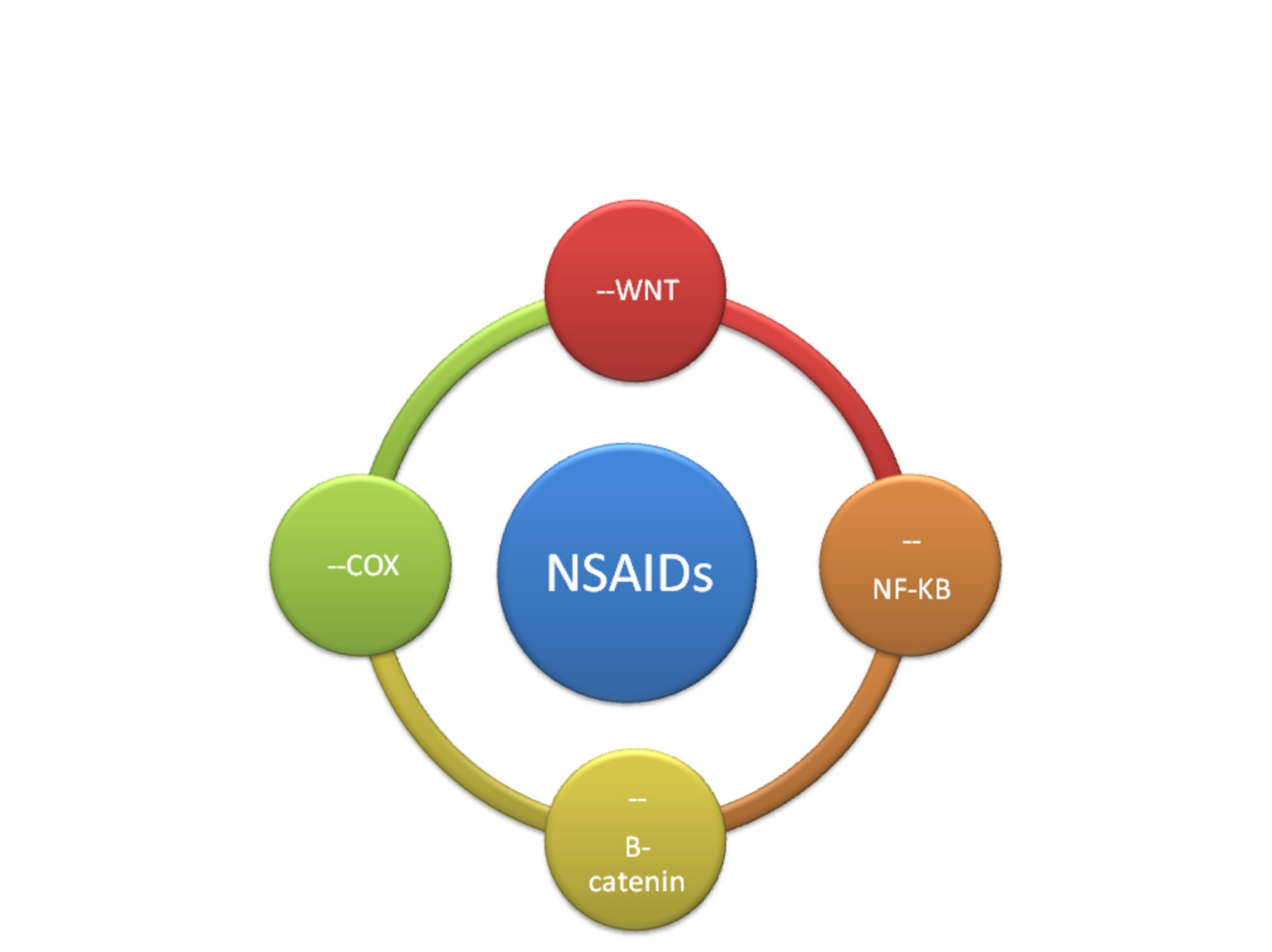
Mechanism of action of NSAIDs in chemoprevention (-- inhibition) NSAIDs, nonsteroidal anti-inflammatory drugs; COX, cyclooxygenase enzyme; B-catenin, catenin beta 1; Wnt, signaling pathway; NF-kB, nuclear factor Kappa light chain enhancer of B cells

The COX non-inhibitory part involves multiple signaling pathways like wnt (signaling pathway), β-catenin, nuclear factor of Kappa light chain enhancer of B cells (NF-κB), p53 (tumor gene), toll-like receptor 4, nitric oxide synthase (NOS-2) and caspases (action for aspirin) sulindac (-p21, p53, Wnt, NF-κB), signal transducer and activator of transcription 3 (STAT3), tumor necrosis factor(TNF-α), IL-1β (interleukins), IL-4, adenosine monophosphate protein kinase (AMPK), proliferating cell nuclear antigen (PCNA), cyclin D1, β-catenin, inducible NOS, COX-2, and p65 (for sulindac with dose 300 mg per day) [10,11,17]. Also, DNA mismatch repair and formation of free radicals through induction of oxidative stress which activates redox responsive signaling leads to stimulation of apoptosis of the cancer cells [12]. The redox formation seems to be prominent and more effective than the COX inhibition pathway. NO-aspirin is nitric oxide-releasing synthase (NOS) along with two COX-2, as well as down-regulated expression of β-catenin and proliferating cell nuclear antigen (PCNA) in colon tumor, all these functions implicated in tumor formation inhibition. It was also reported that aspirin inhibits metastasis by inhibiting Toll-like receptors (Figure 2). Use of this uniquely synthesized formula of NO-and or H2S, release aspirin with protective function to the epithelium of the stomach. This will ensure gastrointestinal safety and serves as a better cancer-preventive option than the parental molecules. Studies report that NO-aspirin suppresses both invasive and non-invasive carcinoma [15].

Treatment with aspirin using a dose of 81-325 mg or more can reduce the 20-year risk of CRC by 24% and decrease cancer-associated mortality by 35% (randomized trial study) [11]. Also, the antiplatelet effect of aspirin has proved to have an anti-tumor impact since the low dose of aspirin given to prevent the cardiovascular accident has shown to decrease the incidence of adenoma of CRC [13].

The chemopreventive effect of NSAIDs (5-ASA, mesalazine, sulindac) has been studied and found that these drugs, when prescribed to a patient with IBD, showed a decrease in colon cancer by 62%; these findings provoked considerable interest in permanently adding 5-ASA or its analogs to the protocol treatment of UC patients. These anti-tumor effects, however, could only be achieved with large doses as mentioned before, and when administered systemically may have a questionable safety profile, as the drug may lead to severe complications.

Side Effects of Long-Term Usage of NSAIDs 

Long-term use of aspirin and NSAIDs in large doses can cause several side effects, which can prevent its use. COX-1 enzyme and its products like prostaglandin 1 (PG1) maintains the integrity of the mucosal layer of the gastrointestinal system. Inhibition of this protective effect by using these drugs can cause erosive gastritis, peptic ulcer, anemia due to chronic bleeding, and peptic ulcer perforation which may even cause death form acute hemorrhage. The bleeding is also aggravated by the antiplatelet effect of aspirin [14]. Adding to this, its impact on the kidney causing interstitial nephritis and chronic kidney failure.

A possible association between the use of NSAIDs and the onset or flare-up of IBD has been suggested, using clinical evaluation and measurement of fecal calprotectin (an indicator of intestinal inflammation) demonstrate that using non-selective NSAIDs associated with 17% to 28% of disease relapse; however, lack of evidence to find a definite conclusion [24].

Selective COX-2 inhibitors like celecoxib show less gastrointestinal tract (GIT) toxicity compared to non-selective NSAIDs, with better control of IBD's extraintestinal manifestations (osteoarthritis). However, their long-term use carries the risk of cardiovascular complications like heart failure, hypertension, myocardial infarction, and stroke. These cardiovascular complications are more common with selective COX-2 than non-selective NSAIDs. Thromboxane is a potent vasoconstrictor formed by the COX-1 enzyme and is not inhibited by COX-2 inhibitors, while prostacyclin, which is a vasodilator formed by COX-2 is inhibited by COX-2 inhibitors, this doubles the vasoconstrictor effect of selective COX-2 inhibitors [25]. All these complications can limit the use of NSAIDs as chemopreventive drugs for cancer colon in IBD patients.

## Conclusions

IBD is a sum of diseases characterized by alteration in the immune system leading to abnormal response, causing inflammation of gastrointestinal layers. Most of the inflammatory mediators implicated in this process also help in the progression of colon cancer. Several anti-inflammatory drugs are used in the treatment of acute attack and/or permanent remission of the disease. The mechanisms of these drugs to do so are COX-2 inhibitory and COX-2 non-inhibitory effect. Most of the NSAIDs and aspirin are needed to be taken for an extended period to reach a promising result, which puts the patient at risk for many complications of long-term usage like anemia, gastrointestinal ulceration, myocardial infarction, stroke, bleeding, kidney dysfunction and even flare of IBD. That's why more studies are needed in the future to know the lowest effective dose with the more local action and least systemic absorption.
